# Multiparametric Flow System for the Automated Determination of Sodium, Potassium, Calcium, and Magnesium in Large-Volume Parenteral Solutions and Concentrated Hemodialysis Solutions

**DOI:** 10.1155/JAMMC/2006/47627

**Published:** 2006

**Authors:** Mariela Pistón, Isabel Dol, Moisés Knochen

**Affiliations:** Departamento Estrella Campos Facultad de Química Universidad de la República Avenida Gral. Flores 2124 Montevideo 11800 Uruguay

## Abstract

A multiparametric flow system based on multicommutation and binary sampling has been designed for the automated determination of sodium, potassium, calcium, and magnesium in large-volume parenteral solutions and hemodialysis concentrated solutions. The goal was to obtain a computer-controlled system capable of determining the four metals without extensive modifications. The system involved the use of five solenoid valves under software control, allowing the establishment of the appropriate flow conditions for each analyte, that is, sample size, dilution, reagent addition, and so forth. Detection was carried out by either flame atomic emission spectrometry (sodium, potassium) or flame atomic absorption spectrometry (calcium, magnesium). The influence of several operating parameters was studied. Validation was carried out by analyzing artificial samples. Figures of merit obtained include linearity, accuracy, precision, and sampling frequency. Linearity was satisfactory: sodium, $r^{2}>0.999$ (0.5–3.5 g/L), potassium, $r^{2}>0.996$ (50–150 mg/L), calcium, $r^{2}>0.999$ (30–120 mg/L), and magnesium, $r^{2}>0.999$ (20–40 mg/L). Precision ($s_{r}$, %, n=5) was better than 2.1%, and accuracy (evaluated through recovery assays) was in the range of 99.8%–101.0% (sodium), 100.8–102.5% (potassium), 97.3%–101.3% (calcium), and 97.1%–99.8% (magnesium). Sampling frequencies ($h^{-1}$) were 70 (sodium), 75 (potassium), 70 (calcium), and 58 (magnesium). According to the results obtained, the use of an automated multiparametric system based on multicommutation offers several advantages for the quality control of large-volume parenteral solutions and hemodialysis concentrated solutions.


Hindawi Publishing Corporation
Journal of Automated Methods and Management in Chemistry
Volume 2006, Article ID 47627, Pages 1–6
DOI 10.1155/JAMMC/2006/47627

### Multiparametric Flow System for the Automated Determination of Sodium, Potassium, Calcium, and Magnesium in Large-Volume Parenteral Solutions and Concentrated Hemodialysis Solutions

**Mariela Pistón, Isabel Dol, and Moisés Knochen**

*Departamento Estrella Campos, Facultad de Química, Universidad de la República, Avenida Gral. Flores 2124, 11800 Montevideo, Uruguay*

Received 31 January 2006; Revised 31 May 2006; Accepted 5 June 2006

A multiparametric flow system based on multicommutation and binary sampling has been designed for the automated determination of sodium, potassium, calcium, and magnesium in large-volume parenteral solutions and hemodialysis concentrated solutions. The goal was to obtain a computer-controlled system capable of determining the four metals without extensive modifications. The system involved the use of five solenoid valves under software control, allowing the establishment of the appropriate flow conditions for each analyte, that is, sample size, dilution, reagent addition, and so forth. Detection was carried out by either flame atomic emission spectrometry (sodium, potassium) or flame atomic absorption spectrometry (calcium, magnesium). The influence of several operating parameters was studied. Validation was carried out by analyzing artificial samples. Figures of merit obtained include linearity, accuracy, precision, and sampling frequency. Linearity was satisfactory: sodium, $r^2 > 0.999$ (0.5–3.5 g/L), potassium, $r^2 > 0.996$ (50–150 mg/L), calcium, $r^2 > 0.999$ (30–120 mg/L), and magnesium, $r^2 > 0.999$ (20–40 mg/L). Precision ($s_r$, %, $n = 5$) was better than 2.1%, and accuracy (evaluated through recovery assays) was in the range of 99.8%–101.0% (sodium), 100.8–102.5% (potassium), 97.3%–101.3% (calcium), and 97.1%–99.8% (magnesium). Sampling frequencies ($h^{-1}$) were 70 (sodium), 75 (potassium), 70 (calcium), and 58 (magnesium). According to the results obtained, the use of an automated multiparametric system based on multicommutation offers several advantages for the quality control of large-volume parenteral solutions and hemodialysis concentrated solutions.

Copyright © 2006 Mariela Pistón et al. This is an open access article distributed under the Creative Commons Attribution License, which permits unrestricted use, distribution, and reproduction in any medium, provided the original work is properly cited.

### 1. INTRODUCTION

Large-volume parenteral solutions are injections widely used in hospitals for treatment of a wide array of conditions. They are aqueous solutions containing one ore more salts (mainly chlorides) of sodium, potassium, calcium, and magnesium, and may contain other substances such as dextrose or lactate.

Hemodialysis concentrated solutions are qualitatively similar in composition to large-volume parenterals but the concentration of the salts is higher. They should be carefully diluted before use to obtain the final solutions.

From the analytical point of view, both types of substances are similar and, for quality control purposes, the same analytical techniques are employed. According to pharmacopeial monographs [1, 2], sodium, potassium, calcium, and magnesium should be determined by flame atomic spectrometry (either absorption or emission for Na and K, and absorption for Ca and Mg).

Despite the fact that the measurement methods are straightforward, the determination of these four metals is not exempt from difficulties. Atomic spectrometry is a technique for trace levels (mg/L), thus samples should be diluted several times to reach analytical levels. For instance, sodium in large-volume parenterals is formulated at concentrations in excess of 3 g/L, while in hemodialysis concentrate solutions it can reach over 100 g/L. Thus a 10000- or even 100000-fold dilution may be necessary to obtain a sample in the mg/L range. This entails considerable manipulation and glassware usage. The risk of contamination or human error is high, as is the uncertainty added in cascade dilutions.

Laboratory automation [3] provides increased productivity and minimizes glassware usage, and also reducing

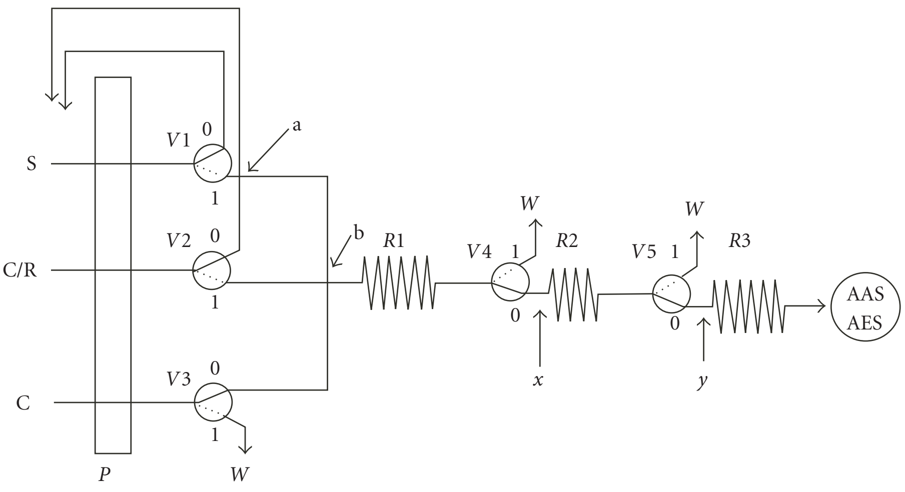

Figure 1: Schematic diagram of the flow system. *P*: peristaltic pump. *V*1, *V*2, *V*3, *V*4, *V*5: solenoid valves. *R*1, *R*2, *R*3: mixing coils. AAS/AES: atomic absorption/emission spectrometer.

drastically the risk of sample contamination. Flow techniques such as flow-injection analysis [4–6] are being used increasingly in quality control in the pharmaceutical laboratory. Nevertheless, little has been published in terms of automation of analysis of large-volume parenteral solutions or hemodialysis concentrates.

Flow-injection analysis has been used successfully for the automation of the determination of sodium, potassium, calcium, and magnesium in parenteral solutions [7]. FIA turned out to be satisfactory for the purpose in terms of precision, accuracy, and sampling frequency. However, given the differences in concentration between elements, different dilutions are required, thus different system configurations should be used for each element, that is, different loop volumes, flow rates, and so forth.

Consequently, research was undertaken with the goal of designing a multiparametric system. Such a system should be able to process samples and standards for each analyte changing only the operating parameters under software control and with a minimum of physical modifications on the system itself.

Multicommutated flow analysis (MCFA) [8, 9] is an emerging flow-analysis technique based on the use of separate solenoid valves operated individually in binary fashion (i.e., on-off) allowing the design of flexible flow networks. The characteristics of this technique seem ideal for the implementation of a multiparametric system.

Multicommutation has been applied successfully to the determination of dextrose in the analysis of large-volume parenteral and hemodialysis solutions [10].

The work presented in this paper refers to the design and evaluation of an automated multiparametric system based on multicommutated flow analysis for the determination of sodium, potassium, calcium, and magnesium.

### 2. MATERIALS AND METHODS

### 2.1. Flow system

The flow system (Figure 1) consisted of a Gilson (Villiers-le-Bel, France) Minipuls 2 multichannel peristaltic pump, and five NResearch (West Caldwell, NJ, USA) 161T031 three-way 12-volt solenoid valves. Connections were made with 0.8 mm (internal diameter) FEP tubing, which was also used for winding the doubly helical mixing coils.

Detection was carried out by means of a Perkin Elmer (Norwalk, Conn, USA) model 380 atomic absorption spectrometer, used in the emission mode (Na, 589.0 nm; K, 766.5 nm) or in the absorption mode (Ca, 422.7 nm; Mg, 285.2 nm). Air-acetylene flame and a 10 cm burner were used. The latter was rotated 45° for emission measurements. Hollow cathode lamps (Photron, Narre Warren, Australia) were used for AA measurements.

Solenoid valves were controlled from an IBM-compatible personal computer by means of a CoolDrive 161D5X12 driver (NResearch) connected to five data bits (D0–D4) of the LPT1 printer port.

Analog data from the spectrophotometer were acquired via the recorder output by means of a 12-bit analog-to-digital (A/D) interface (Measurement Computing, Middleboro, Mass, USA, model CIO-DAS-08Jr) installed in the ISA bus of the computer.

The system was controlled with a program compiled in QuickBASIC 4.0 language running under the MS-DOS operating system. The program controlled the solenoid valves providing the appropriate timing and acquired data via the A/D interface. The data (absorbance) were plotted in real time on the screen and stored on hard disk for later processing.

### 2.2. Reagents

Sodium chloride, potassium chloride, calcium chloride dihydrate, magnesium chloride hexahydrate, lactic acid, and acetic acid of analytical reagent grade were used as received. The purity of the calcium and magnesium salts was checked chelatometrically and the results were taken into account in the calculations. Lanthanum oxide 99.9% (Sigma) was used as releasing agent.

Water was distilled in an all-glass still (Aquatron A-4000, Bibby Sterilin, Staffordshire, UK) and further purified in a Millipore (São Paulo, Brazil) Simplicity 185 water purifier.

### 2.3. Artificial samples

For the recovery assays, synthetic samples of several formulations were prepared by exact weighing and dilution of each of the ingredients. These formulations were representative of several large-volume parenteral solutions and concentrate hemodialysis solutions found both in the United States Pharmacopeia (USP) and in the market:

(i) Ringer's injection (USP);
(ii) lactated ringer's and dextrose injection (USP);
(iii) hemodialysis concentrated solution with dextrose (containing NaCl, KCl, sodium acetate trihydrate, $CaCl_2 \cdot 2H_2O$, $MgCl_2 \cdot 6H_2O$, dextrose, water);
(iv) acidic hemodialysis concentrated solution with dextrose (containing NaCl, KCl, acetic acid, $CaCl_2 \cdot 2H_2O$, $MgCl_2 \cdot 6H_2O$, dextrose, water).

### 2.4. Operation of the system

The same system was used for the determination of the four analytes, however the operation of the valves was different for each one. The R/C line was fed either with water (sodium, magnesium), 3% (m/v) La solution (calcium determination), or 15 g/L Na solution (potassium determination). These two reagents were added to correct for chemical and ionization interferences, respectively.

The control and data-acquisition program featured a parameters menu where all operating parameters (such as the time involved in each step, the number of segments used in the sampling, etc.) were displayed and could be readily modified as necessary. A specific timing diagram was devised for each analyte after a series of optimization experiments.

Samples were introduced to the system by binary sampling [11]. At the beginning of the analytical cycle, several short segments of sample and water (or reagent) were inserted in the sequence S−R−S−R· · · , where S was the sample and R either water or reagent. For this purpose, valve $V1$ was turned on for periods $t_{v1}$ to insert the sample segments, while valve $V2$ was turned on for periods $t_{v2}$ to insert water (or reagent) segments. In this context, the reagent was either an ionization buffer (15 g/L Na) for potassium determination or a releasing agent (3% (m/v) La) for calcium determination.

Sample insertion was carried out by alternately turning $V1$ and $V2$ on and off so that at a given time, only one of them was active. Besides, during segment insertion, valve $V3$ was turned on and water was discarded to waste in order to avoid disturbing the flow pattern of the segments.

After the appropriate number of segments was inserted, $V1$ and $V2$ were turned off and $V3$ was also turned off, thus allowing the segments row to be transported by the stream of water (C) towards mixing coil $R1$, where the individual segments intermix and become a single dispersed sample bolus.

Valves $V4$ and $V5$ were used to implement dilution by means of a zone-sampling strategy. After completing the injection process valve, $V4$ was turned on. Thus all of the front and part of the tail of the dispersed sample zone were discarded to waste. After a specified period $t_{d1}$, $V4$ was turned off for a short period $t_{zs1}$, thus resampling a small portion of the tail zone. Afterwards, $V4$ was turned on again for a period $t_{d2}$, discarding to waste the tail of the sample zone. $t_{d2}$ was chosen to ensure that the analyte concentration decreased to negligible levels. After this time elapsed, $V4$ was turned off again, allowing the flow of water and transporting the resampled zone towards mixing coil $R2$.

In the case of sodium, where higher concentrations were handled, it was necessary to obtain higher dilution rates. This was achieved by using valves $V4$ and $V5$ to implement a dual zone-sampling scheme. The dispersed sample zone was sampled by $V4$ as explained. After $t_{d2}$, the subsample was then dispersed at $R2$ and resampled by $V5$. This valve was turned on for a period $t_{d3}$ in order to discard to waste the front and part of the tail of the dispersed subsample zone, then turned off for a short period $t_{zs2}$ to allow the resampling, and then turned on again for a period $t_{d4}$ to discard the rest of the tail. Afterwards, $V5$ was turned off for the rest of the cycle and the resampled zone was transported to mixing coil $R3$. In the determination of sodium, also $V2$ was turned on during $t_{d4}$ in order to increase the total flow rate to help discarding the tail of the subzone. For the determination of K, Ca, and Mg, $V5$ was not used.

### 2.5. Methods

Standard solutions were prepared in the following ranges: sodium, 1–4 g/L; potassium, 50–150 mg/L; calcium, 30–20 mg/L; magnesium 20–40 mg/L. Calibration curves were obtained by linear regression of peak heights (absorbance) over concentration.

Samples of parenteral solutions were analyzed without dilution; samples of hemodialysis concentrate solutions were diluted by weight 25-fold (acidic hemodialysis concentrate solution with dextrose) or 50-fold (hemodialysis concentrate solution with dextrose) before analysis in order to obtain concentration levels suitable for injection in the flow system.

### 3. RESULTS AND DISCUSSION

### 3.1. Operating parameters

For each analyte, the influence of the following parameters was studied: number of segments (sample and water or reagent), segment size, and discard times ($t_{d1}$ to $t_{d4}$).

Table 1: Final operating parameters of the flow system.

| Parameter | Analyte | | | |
|---|---|---|---|---|
| | Sodium | Potassium | Calcium | Magnesium |
| $t_{v1}$ (s) | 1.5 | 1.4 | 1.3 | 1.5 |
| $t_{v2}$ (s) | 1 | 1 | 1 | 1 |
| No. of segments | 3 | 3 | 3 | 6 |
| $t_{d1}$ (s) | 5 | 9 | 8 | 15 |
| $t_{zs1}$ (s) | 1.5 | 1.5 | 1.5 | 2 |
| $t_{d2}$ (s) | 10 | 10 | 15 | 10 |
| $t_{d3}$ (s) | 6 | 0 | 0 | 0 |
| $t_{zs2}$ (s) | 1.3 | 0 | 0 | 0 |
| $t_{d4}$ (s) | 20 | 0 | 0 | 0 |
| Total time (s) | 51.3 | 47.7 | 51.4 | 62 |
| Sampling frequency ($h^{-1}$) | 70 | 75 | 70 | 58 |

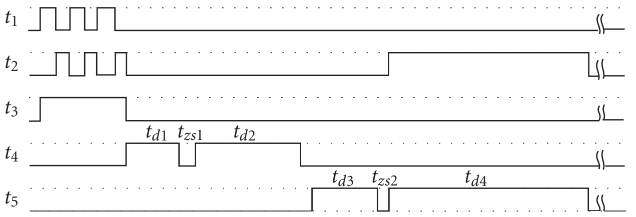

Figure 2: Commutation timing diagram for the determination of sodium.

The number and size of segments were chosen to obtain a sample zone of reasonable volume. Segment size was determined by the activation time of the respective valve ($V1$ or $V2$). The lower limit of this time is given by the electromechanical response time of the valve. According to the manufacturer, the maximum response time for the valves is around 30 milliseconds. Thus it is not recommended to use activation times under 0.3 second as these could increase the dispersion.

Discarding times $t_{d1}$ and $t_{d3}$ were varied in order to ensure that the front and part of the tail of the respective sample zones were discarded to waste. The goal was to resample a low-slope portion of the zone tail, in order to obtain a signal of appropriate height while minimizing the influence of timing uncertainty. Similarly, $t_{d2}$ and $t_{d4}$ were varied to find the optimum values to ensure that the remaining tail of the zone was discarded to waste to a point were its effect became undetectable. Optimum values were chosen based on the height and precision of the signals obtained. The total duration of the analytical cycles was also taken into account in order to maximize the sampling frequency. Best results were obtained with the values shown in Table 1, while an example of a timing diagram can be seen in Figure 2.

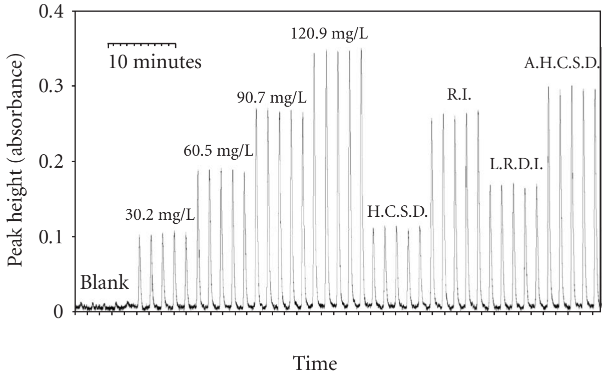

Figure 3: Recording of the signal corresponding to the determination of calcium. Please notice that since the system does not record the baseline between peaks, the time scale is not continuous.

Figure 3 is the plot of a calibration curve and four samples corresponding to a determination of calcium. One of the drawbacks of the proposed system is that during the discarding times $t_{d3}$ and $t_{d4}$, the resampled zone is trapped just past the corresponding valve ($V4$ or $V5$, resp.). This zone does not move while the valve is turned on, adding up to the total duration of the analytical cycle and decreasing the sampling frequency. The use of additional lines pumping water to points $x$ and $y$ (Figure 1) was considered, but this would not have helped as part of the zone is trapped inside the dead volumes within the valve and connection fittings, inaccessible to the additional carrier stream. Even though this drawback could not be overcome, the sampling frequencies obtained were appropriate for the purpose.

Sodium was chosen as ionization buffer instead of the more usual cesium. Sodium is cheaper and given the high concentration in the solution (15 g/L), it was in fact added in large excess and was effective for the purpose. One of the problems that could arise using such high concentrations of sodium is the memory effect that could affect future determinations of this metal. However no noticeable memory effect was found in the determinations of sodium carried out after potassium determinations as long as the C/R line was washed sufficiently with water.

In this context, it should be pointed out that the system used in the present work was basically a prototype intended to show the feasibility of the concept and was designed to work with the minimum amount of parts. However the system could be easily enhanced by adding two more solenoid valves so that separate valves could be available for water, ionization buffer, and releasing agent.

When changing samples, it was necessary to purge from the previous sample the analytical path, especially section a-b (Figure 1). This was attained by switching on valves $V1$ and $V4$ for 25 seconds sending the excess sample to waste, and then off for 25 seconds so that only section a-b remains filled with sample.

The system was capable of handling large-volume parenterals directly without any sample preparation. However hemodialysis concentrate solutions, which have much higher

Table 2: Recovery and precision data for the analysis of four synthetic samples.

| Sample | Analyte | Concentration (g/L) Put | Found | Recovery (%) | Precision $s_r$(%), $n = 5$ |
|---|---|---|---|---|---|
| Ringer's Injection (USP) | Na | 3.130 | 3.12 | 99.8 | 1.0 |
| | K | 0.1395 | 0.143 | 102.5 | 1.6 |
| | Ca | 0.08966 | 0.090 | 100.4 | 1.3 |
| Lactated Ringer's and Dextrose Injection (USP) | Na | 2.970 | 3.00 | 101.0 | 1.3 |
| | K | 0.1395 | 0.141 | 100.8 | 1.8 |
| | Ca | 0.0498 | 0.048 | 97.3 | 0.9 |
| Hemodialysis concentrate solution with dextrose | Na | 125.00 | 125.7 | 100.5 | 1.0 |
| | K | 3.348 | 3.42 | 102.3 | 2.0 |
| | Ca | 1.744 | 1.77 | 101.3 | 2.1 |
| | Mg | 1.350 | 1.35 | 99.8 | 0.9 |
| Acidic hemodialysis concentrate solution with dextrose | Na | 70.43 | 70.7 | 100.5 | 1.5 |
| | K | 2.833 | 2.89 | 101.9 | 2.1 |
| | Ca | 2.615 | 2.60 | 99.4 | 1.3 |
| | Mg | 0.772 | 0.75 | 97.1 | 2.0 |

concentrations of all the analytes, had to be diluted manually once before processing. From the point of view of automation, it would seem desirable to be able to handle these concentrates directly. In fact this is not appropriate because it would pose difficulties in the calibration process. Since the system cannot perform exact dilutions of the samples, standard solutions should be as concentrated as the sample itself, that is, similar to brine. Obviously, this is neither practical nor desirable, thus it was necessary to resort to the manual dilution step.

#### *3.1.1. Dilution factor*

The apparent dilution produced by the system was assessed for each analyte by comparing the concentration that gave a certain signal (peak height) in the multicommutated system, with the concentration necessary to obtain the same signal when pumped directly to the nebulizer at the same flow rate. Results were 900 (sodium), 14 (calcium), 62 (magnesium), and 65 (potassium).

### *3.2. Validation*

#### *3.2.1. Linearity*

Linearity was studied for each analyte for 5-point calibration curves in the working concentration ranges.

Calibrations functions were linear for sodium (0.5–3.5 g/L, $r^2 > 0.999$), potassium (50–150 mg/L, $r^2 > 0.996$), calcium (30–120 mg/L, $r^2 > 0.999$), and magnesium (20–40 mg/L, $r^2 > 0.999$).

#### *3.2.2. Accuracy and precision*

Synthetic samples containing known amounts of sodium, potassium, calcium, and magnesium were analyzed for the four metals using the proposed system. Recoveries (defined as 100* concentration found/known concentration) were calculated and evaluated as a measure of accuracy.

Large-volume parenterals and concentrated hemodialysis solutions are unique samples as their composition is well known and established with detail in the Pharmacopeias. Unlike other pharmaceuticals, there is little margin for variation, for instance unexpected excipients or other concomitants are virtually excluded. Thus these samples are ideal for using the analysis of synthetic samples as validation strategy.

Precision, assessed from the relative standard deviation ($s_r$(%)) of five injections of sample was in the range 0.9%–2.1%. Figures for accuracy and precision for each analyte are presented in Table 2.

Precision in flow systems based on binary sampling depends critically on stability of flow rates and on timing accuracy. Timing of commutation of the solenoid valves is highly accurate; however peristaltic pumps have an inherent pressure ripple that limits precision of the results, especially when very short segments are inserted, comparable in duration with the period of the ripple. To avoid this problem, all valve activation times significant for the operation of the system were set equal or higher than 1 second.

### 4. CONCLUSIONS

The system presented was capable of determining sodium, potassium, calcium, and magnesium in the large-volume parenteral solutions and concentrated hemodialysis solutions. When analyzing synthetic samples the recoveries, precisions, and sampling frequencies found suggested accuracy and precision appropriate for the purpose of quality control.

It is concluded that a multiparametric system based on multicommutation can offer several advantages for the quality control of large-volume parenteral and concentrated hemodialysis solutions.

### ACKNOWLEDGMENTS

The authors thank CSIC-UdelaR (Comisión Sectorial de Investigación Científica de la Universidad de la República) and Programa de Desarrollo de Ciencias Básicas (PEDECIBA, UNDP URU/97/016) for financial support.

### REFERENCES

[1] United States Pharmacopeia, United States Pharmacopeial Convention, Rockville, Md, USA, 26th Revision, 2003.

[2] British Pharmacopoeia (digital version). TSO, London, UK, 2002.

[3] P. B. Stockwell, *Automatic Chemical Analysis*, Taylor & Francis, London, UK, 2nd edition, 1996.

[4] J. Ruzicka and E. Hansen, *Flow Injection Analysis*, John Wiley & Sons, New York, NY, USA, 2nd edition, 1988.

[5] M. Trojanowicz, *Flow Injection Analysis: Instrumentation and Applications*, World Scientific, Singapore, Republic of Singapore, 2000.

[6] J. Martínez Calatayud, *Flow Injection Analysis of Pharmaceuticals; Automation in the Laboratory*, Taylor & Francis, London, UK, 1996.

[7] I. Dol, G. Espínola, and M. Knochen, "Automatic determination of metals in parenteral solutions by FIA-AS," *Atomic Spectroscopy*, vol. 20, no. 1, pp. 39–43, 1999.

[8] M. Catalá Icardo, J. V. García Mateo, and J. Martínez Calatayud, "Multicommutation as a powerful new analytical tool," *Trends in Analytical Chemistry*, vol. 21, no. 5, pp. 366–378, 2002.

[9] F. R. P. Rocha, B. F. Reis, E. A. G. Zagatto, J. L. F. C. Lima, R. A. S. Lapa, and J. L. M. Santos, "Multicommutation in flow analysis: concepts, applications and trends," *Analytica Chimica Acta*, vol. 468, no. 1, pp. 119–131, 2002.

[10] M. Knochen, M. Pistón, L. Salvarrey, and I. Dol, "A multicommuted flow system for the determination of dextrose in parenteral and hemodialysis concentrate solutions," *Journal of Pharmaceutical and Biomedical Analysis*, vol. 37, no. 4, pp. 823–828, 2005.

[11] B. F. Reis, M. F. Giné, E. A. G. Zagatto, J. L. F. C. Lima, and R. A. Lapa, "Multicommutation in flow analysis. Part 1. Binary sampling: concepts, instrumentation and spectrophotometric determination of iron in plant digests," *Analytica Chimica Acta*, vol. 293, no. 1-2, pp. 129–138, 1994.

